# Supplementary material for: High-frequency magnetohydrodynamic waves with substantial energy in the solar polar corona
Source: Natl Sci Rev. 2026 Jun 24;13(14):nwag370. doi: 10.1093/nsr/nwag370 (PMC13386508; doi:10.1093/nsr/nwag370)
Supplement: nwag370_Supplemental_File [file nwag370_supplemental_file.pdf]

National Science Review
: supplementary,
doi: https://doi.org/

High-Frequency Magnetohydrodynamic Waves with Substantial Energy in the Solar Polar Corona: Supplementary Information

Additional backgrounds on propagating kink waves in coronal open-field regions

Kink waves are one of the most extensively studied waves in the solar atmosphere. In magnetic flux tubes, kink waves are defined as magnetoacoustic waves with the azimuthal wave number $m = 1$ [1]. Under the thin flux tube approximation, such waves have been shown to be nearly incompressible transverse waves, with the velocity and magnetic field perturbations perpendicular to the wave vector. This makes kink waves share very similar properties to the Alfvén waves, and therefore kink waves are often called Alfvénic waves [2–4]. In observations, kink waves are relatively easy to be detected as they manifest as lateral oscillations of the whole flux tube. Such waves have been reported in various solar structures, including coronal loops, plumes, magnetic bright points, spicules, jets, and prominences [5–13].

In this work, we focus on propagating kink waves (PKWs) in coronal open-field regions, particularly within polar plumes. These waves can be detected through two observational approaches. The first relies on spectroscopic measurements. The Coronal Multi-Channel Polarimeter (CoMP; [14]) provides spectra of the Fe XIII 1074.7 nm line across the off-limb corona. The derived Doppler velocity maps reveal ubiquitous outward-propagating fluctuations [15], which were later interpreted as kink waves [16]. The second approach uses extreme-ultraviolet (EUV) imaging, which allows direct detection of transverse motions in plumes above the solar limb [3,17–23]. This work applied the second approach.

PKWs have drawn considerable attention since their discovery because they are a pervasive coronal phenomenon that can serve as powerful diagnostic tools for inferring plasma parameters, particularly the magnetic field [24–27]. Moreover, these waves are believed to play an important role in coronal heating and solar wind acceleration, especially within coronal holes [3, 28–33]. The latter aspect is the central focus of this study.

Quantifying PKW's contribution in coronal heating and solar wind acceleration requires reliable measurements of wave amplitudes and corresponding energy fluxes. Spectroscopic observations typically yield velocity amplitudes below 1 km s^{-1} and energy fluxes less than 1 W m^{-2} [15,34,35], which are much lower than the $\gtrsim$ 100 W m^{-2} required to sustain coronal-hole heating [36,37]. In contrast, imaging observations report larger amplitudes of 10–20 km s^{-1} [3,17,21]. This discrepancy in amplitude measurements is largely attributed to line-of-sight averaging effects in spectroscopic studies (see simulations by [38–40]), while imaging data can resolve individual plume motions and thus provide more reliable amplitudes (e.g., [3,29]). Although energy-flux estimates from imaging vary depending on the adopted methods and assumptions, most studies report values below $\sim$100 W m^{-1} (e.g., 9-24 W m^{-2} in [17]; 28-71 W m^{-2} in [20]; and 50-80 W m^{-2} in [28]). Nevertheless, these estimates may still underestimate the true flux because high-frequency PKWs are likely underdetected. As mentioned in the main text, most previously reported events have periods longer than 100 s, limited by the cadence of earlier datasets (e.g., 12 s for SDO/AIA and 33.5 s for CoMP). In contrast, high-cadence chromospheric observations frequently reveal kink waves in spicules with periods below 100 s [6,10,41]. Recent EUV observations have also identified numerous high-frequency kink oscillations in small coronal loops [42–46], enabled by the high spatial and temporal resolution of Solar

·.

© The Author(s) . Published by Oxford University Press on behalf of China Science Publishing & Media Ltd. This is an Open Access article distributed under the terms of the Creative Commons Attribution License (http://creativecommons.org/licenses/by/4.0/), which permits unrestricted use, distribution, and reproduction in any medium, provided the original work is properly cited.

Orbiter/EUI. This naturally raises the question: can high-frequency propagating kink waves also be abundantly detected in coronal plumes using Solar Orbiter/EUI observations?

Another key question concerning PKWs is their origin. Theoretical and numerical studies have proposed three primary mechanisms. First, turbulent convective motions in the photosphere can continuously buffet flux-tube footpoints, exciting kink waves that propagate upward [8,47]. Although a fraction of these waves may be reflected or dissipated below the transition region, they can still carry substantial energy into the corona [48]. Second, mode conversion from photospheric p-modes can also produce kink waves [49,50], consistent with the observed power enhancement near $\sim$3.5 mHz in coronal wave power spectra [15,28,51,52]. Third, interchange reconnection between open and closed magnetic fields can naturally generate Alfvénic perturbations that propagate along newly-formed open field lines [53–57]. However, direct observations of these generation mechanisms remain very limited (for a review see [4]).

As mentioned above, the wave power spectrum offers valuable clues to the underlying generation processes. Morton et al. [52] analyzed power spectra derived from spectroscopic observations of open-field coronal regions obtained with DKIST/Cryo-NIRSP. They attributed the enhanced power at distinct frequency bands to different physical origins and further estimated their relative contributions.

In this study, we used high-cadence and high-resolution Solar Orbiter/EUI observations, combined with power-spectral analysis, to address the following questions: (1) How abundant are high-frequency kink waves propagating in coronal plumes within coronal holes? (2) How significant is their contribution to the coronal energy budget compared with that of low-frequency waves? (3) What mechanisms can be likely responsible for generating these high-frequency transverse waves? These questions were responded in the 'Results' and 'Discussion' sections of the main text.

Examples of detected wave events

In Figure S1 and Figure S2, we present several examples of wave events detected in EUI data with NUWT. The identified oscillating threads provide us with time series of transverse displacements, which are further used to generate Fourier power spectra. The power peaks above the 95% significance level help determine the valid wave events, which can be at most 4 for one individual thread. Not all threads correspond to valid wave events, as shown in panels (f1) and (f2) in Figure S1.

Height variation of wave parameters

In this section, we examined the height-dependent variation of the wave parameters in EUI observations. Figure S3 shows number of oscillating threads (corresponding to blue dashed lines in Figure 1E in the main text) detected by NUWT at each heights. The result indicates that the thread number decreases with height. We note that each thread could correspond to more than one wave events as these threads often show a multi-frequency nature.

Figure S4 shows box plots constructed from the waves identified in EUI observations. Each box corresponds to one of the 17 slits used to generate the time–distance maps. In each box, the central line represents the median value, while the lower and upper edges denote the 25th (Q1) and 75th (Q3) percentiles, respectively. Consistent with prior studies [21,22], the median wave period exhibits a slight decrease with height. In contrast, the amplitude shows an opposite trend: while the increase in displacement amplitude is subtle, the corresponding velocity amplitude demonstrates a more pronounced upward trend.

The height-dependent trends of wave parameters revealed here provide insights into the propagation and evolution of kink waves in the open corona. The period appears to decrease slightly with height. This trend could be attributed to wave reflection, since long-period waves are more susceptible to reflection. Such waves have long wavelengths, which can lead to substantial variations in phase speed over a single wavelength. Reflection may thus arise from gradients in the phase speed [58]. Another contributing factor could be that the SNR generally decreases with height as the intensity weakens, making it more difficult to track oscillating plumes over long durations. This could also hinder the detection of long-period waves, producing the observed statistical decrease in periods. Additionally, the period trend flattens above $\sim$15 Mm, which could be consistent with the prediction of "uniturbulence" theory that wave damping is largely independent of period [59].

The increase in velocity amplitude with height is a well-established consequence of vertical density stratification [32,60]. Under the assumption of nearly constant wave energy flux, the velocity amplitude is shown to be proportional to $\langle\rho\rangle^{-1/4}$; hence, decreasing density with

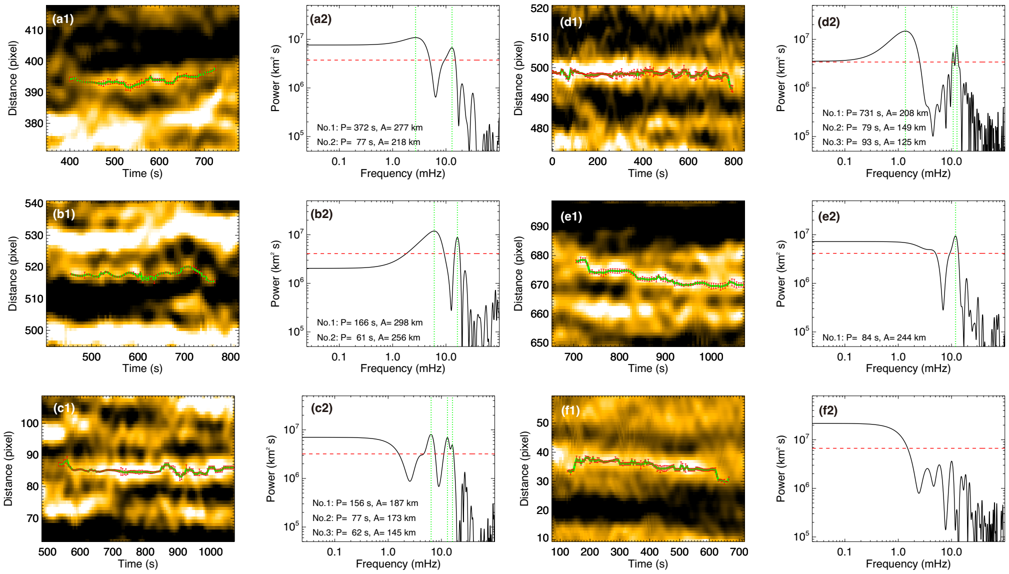

Figure S 1. Six examples of detected oscillating threads at a height of 11.8 Mm. For each example, the left panel (a1, b1, c1, d1, e1, f1) shows a zoomed-in view of the time–distance map, with the thread position marked by green solid lines and red error bars. The right panel (a2, b2, c2, d2, e2, f2) displays the corresponding Fourier power spectrum (black solid line) derived from the transverse displacement time series. In each Fourier spectrum, the red dashed line indicates the 95% significance level, and green dotted lines denote frequencies or periods with significant power, which are identified as valid wave events. The dominant period and displacement amplitude for each wave event are labeled in the bottom-left corner.

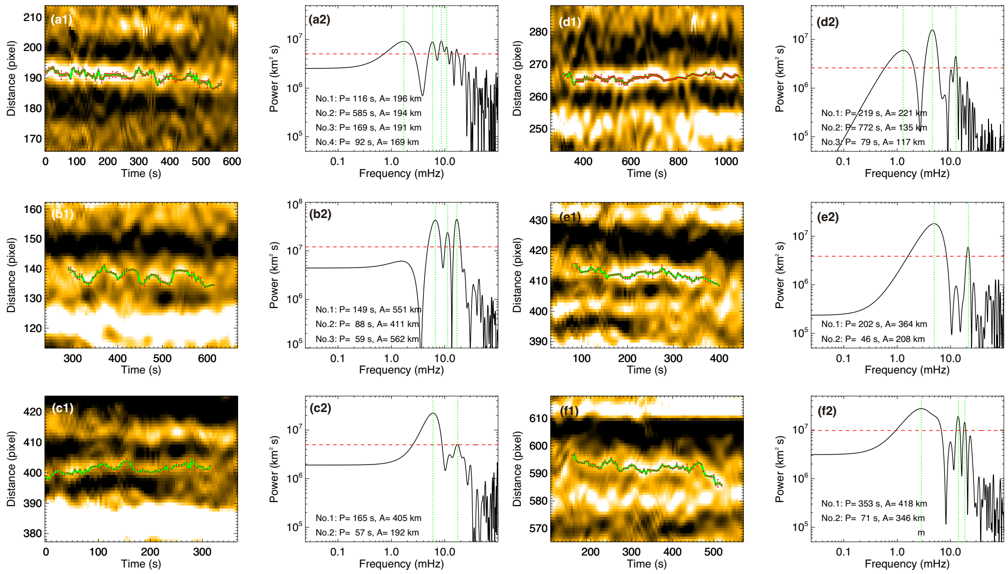

Figure S 2. Similar to Figure S1 but at a height of 34.9 Mm.

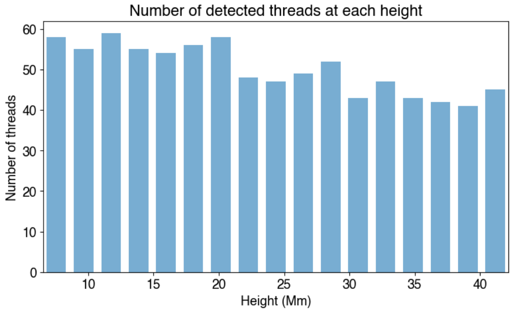

Figure S 3. The number of detected threads from time-distance maps generated at different heights.

height naturally leads to increasing amplitudes. Wave damping mechanisms, such as resonant absorption [61,62], can further modulate this behavior. The combined effects of density stratification and damping were explored with three-dimensional MHD simulations in [32], which found that damping is less significant at lower heights compared to stratification. In their setup, the velocity amplitude increases with height up to ~45 Mm before decreasing at higher altitudes, consistent with the trends observed here. Extending the analysis to greater heights could provide insights into wave damping. However, in the current dataset, the SNR becomes quite low above ~40 Mm, and therefore we chose not to extend the wave identification to higher regions.

We further analyzed the power spectra across four distinct height ranges, with each range containing 536–614 wave events to ensure robust statistical analysis. The results are presented in Figure S5. No evident power damping with height is detected, indicating that resonant absorption damping is not significant relative to the stratification effect in the corona below 42 Mm. This is consistent with previous works demonstrating weak damping in the lower open-field corona [63,64]. Notably, the amplitude enhancement is most pronounced in the 10–20 mHz high-frequency band. By contrast, power in the low-frequency range (2–4 mHz) shows a slight decrease with height. A plausible interpretation is that low-frequency waves have longer wavelengths, making them more susceptible to reflection in the vertically stratified atmosphere [58].

The absence of clear height-dependent damping can also be explained by two additional factors. First, it may be related to statistical bias at different heights. At greater heights, our wave detection preferentially identifies shorter-period events with larger velocity amplitudes (see Figure 4(B) in the main text and Figure S4), which could mask the expected damping trend. Second, additional high-frequency waves may be

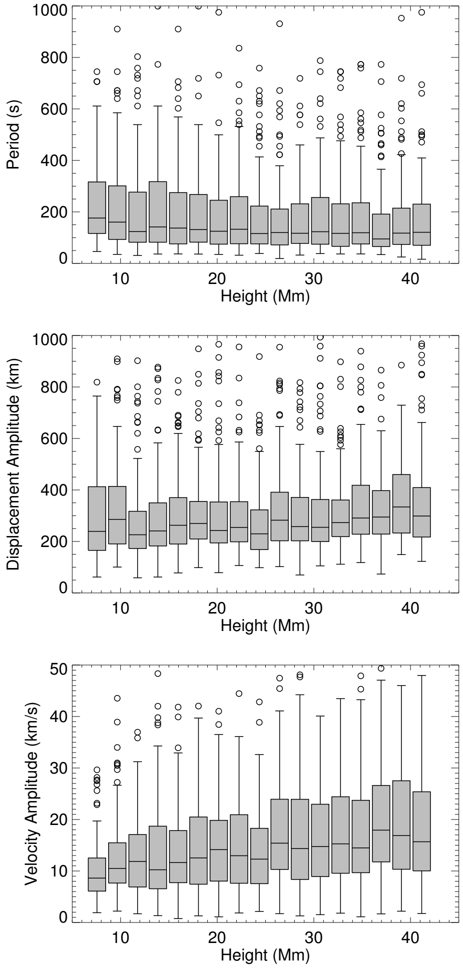

Figure S 4. Box plots showing the height variation of wave period, displacement amplitude, and velocity amplitude derived from EUI observations. The whiskers extend to the most extreme data points within 1.5 times the inter-quartile range (IQR = Q3 – Q1), and data points beyond this range are plotted individually as circles, representing outliers.

continuously generated with increasing height via coronal dynamical processes such as interchange magnetic reconnection and turbulent cascading [56]. Furthermore, nonlinear mechanisms such as uniturbulence can lead to energy cascade without following a $1/f$ damping dependence [59], which may also contribute to the apparent absence of frequency-dependent damping in the observed height range.

To achieve a more rigorous assessment, future work should incorporate height-dependent density diagnostics to account for the vertical density stratification and compute the intrinsic wave energy flux at different frequencies, rather than relying on projected velocity amplitudes alone. It would also be valuable to track individual wave events across heights along single plume

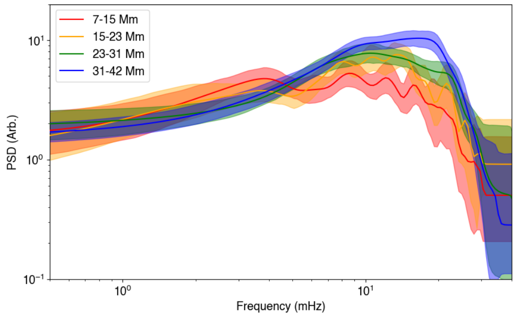

Figure S 5. Power spectra for four different height ranges observed by EUI. The shaded regions indicate the 95% confidence intervals (CIs). The scatter points corresponding to individual wave events are omitted for a better visualization.

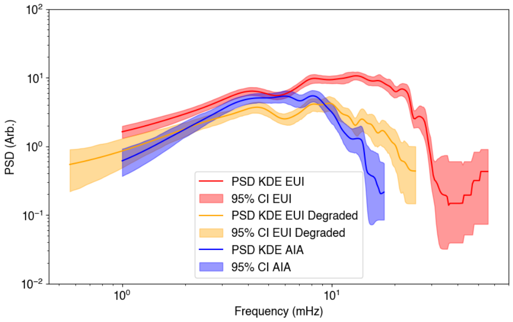

Figure S 6. Similar to Figure 4(C) of the main text but including the degraded EUI data (orange curve) to examine the effect of spatial and temporal resolution. Note that the scatter points (PSDs for individual wave events) are omitted for a better visualization.

structures to reduce sampling biases. Furthermore, future investigations using higher signal-to-noise ratio data and extended height coverage could also enable direct detection of wave power damping with altitude, allowing quantitative constraints on wave damping lengths.

Instrumental effects on the wave power spectra: spatio-temporal resolution and jitter

To further assess the influence of instrumental cadence and resolution on wave detection and PSD estimation, we degraded the EUI data to match AIA's spatial resolution and cadence, and repeated the same analysis procedures. The resulting PSD (orange curve in Figure S6) shows a noticeable power reduction above 10 mHz, confirming that observational resolution and cadence strongly influence the measured power spectra. While the PSD derived for degraded EUI data becomes more comparable to the AIA spectrum, some differences remain between them, which is as expected due to other instrumental distinctions such as wavelength response, optical design, noise characteristics, and viewing angles.

We also evaluated the possible impact of residual image jitter on the PSD estimation. Although the EUI data were largely corrected for jitter using a cross-correlation method, minor residual effects could still produce spurious apparent motions, which might lead to error detection of wave events. Considering that the residual jitter should be not very large, the events with small displacement amplitudes are more likely affected by this. Thus we recalculated the PSDs (shown in Figure S7) by imposing different lower threshold on the measured displacement amplitude A: $A > 0.5$ pixel (orange) and $A > 1$ pxiel (yellow). We can notice that the high-frequency portions of the spectra ($\gtrsim$10 mHz) are virtually identical across different thresholds, demonstrating that the high-frequency power is robust against potential jitter artifacts. However, the proportion of the total integrated power of the high-frequency part relative to the low-frequency part will decrease accordingly. This is not surprising, as high-frequency fluctuations are more common among low-displacement amplitude wave events (see Figure 4(A) of the main text). Even so, for the case where $A > 1$ pixel, the integrated power of the high-frequency part is comparable to that of the low-frequency part. Moreover, it can also be known from the TD maps (Figure 1(E) and Figure 2(D) of the main text) that the influence of jitter is not significant because there are not coherent transverse motions, which are expected signatures of jitter.

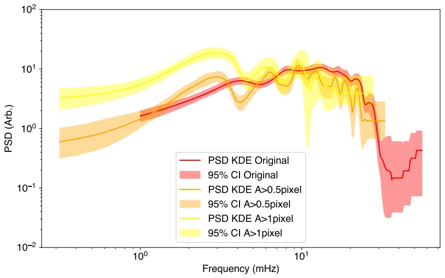

Figure S 7. Comparison of power spectra for all EUI wave events (red) and subsets with displacement amplitudes $A > 0.5$ pixel (orange) and $A > 1$ pixel (yellow).

Calculation of power spectral density

The calculation of power spectral density (PSD) follows the method described in [28]. We first calculated mean square velocity for each wave event as

$$\mathrm{MS}_{v,i} = T_i v_i^2 / T_{\mathrm{tot}} ,$$

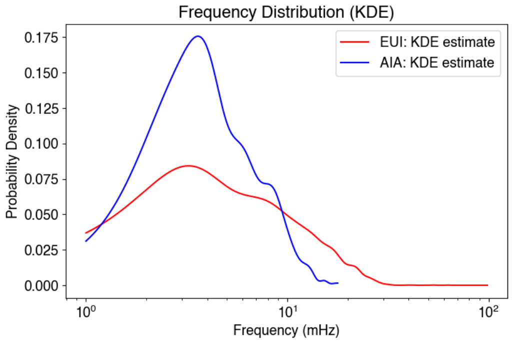

Figure S 8. Occurrence probability density of frequency for EUI (red) and AIA (blue) results.

where T_i and v_i is the duration and velocity amplitude of the i-th event detected by Auto-NUWT [20], and T_{tot} is total duration of the analyzed image sequences. The next step is to obtain a smooth estimate of the distribution of oscillation frequencies, for which we applied the Gaussian kernel density estimation (KDE). The optimal bandwidth parameter was determined by grid search using cross-validation. The bandwidth parameter of the Gaussian kernel was searched within 0.1–10 mHz using `sklearn.model_selection.GridSearchCV` in `scikit-learn v1.5`, corresponding to the characteristic frequency resolution of the EUI time series (5 s cadence and a total duration of 1075 s). The resulting KDE provides an estimate of the normalized occurrence probability of frequencies

$$\hat{p}(f) = \exp[\mathrm{KDE}(f)] \, .$$

The results for EUI and AIA are shown in Figure S8. We note that similar method is also applied to obtain the distribution of amplitudes and periods, as shown in Figure 3 of the main text.

The PSD for each oscillation was then defined as

$$\mathrm{PSD}_i = \hat{p}(f_i)\mathrm{MS}_{v,i} \, ,$$

representing the energy distribution of velocity fluctuations across frequencies, as shown as scatter points in Figure 4(C) of the main text.

To obtain a smooth, bias-corrected PSD curve, we further applied a radial basis function (RBF) kernel regression model [65]. The RBF kernel width parameter γ was optimized within the range 0.1–100 mHz^{-2} using grid search, ensuring a balance between smoothness and local adaptability in the PSD fitting. Bootstrap resampling ($N_{bs} = 1000$) was performed to estimate the uncertainty of the regression. For each resampled dataset, a new KDE was fitted, and the smoothed PSD was predicted at logarithmically spaced frequencies between a suitable range (for EUI, it is $[10^{-3}, 10^{-1}]$ Hz; for AIA, it is $[10^{-3}, 10^{-1.75}]$ Hz). The mean PSD (solid curves in Figure 4(C) of the main text) was computed as

$$\langle \mathrm{PSD}(f) \rangle = \frac{1}{N_{bs}} \sum_{j=1}^{N_{bs}} \mathrm{PSD}_j(f) \, .$$

The 95% confidence intervals (CIs; shaded regions in Figure 4(C) of the main text) were obtained from the 2.5th and 97.5th percentiles of the bootstrap distribution, corresponding to the central 95% probability mass of the estimated PSD.

REFERENCES

1. Roberts, Bernard. *MHD waves in the solar atmosphere*, (Cambridge University Press2019).
2. Goossens M, Terradas J, Andries J *et al.* On the nature of kink MHD waves in magnetic flux tubes. *Astronomy and Astrophysics* 2009; **503**: 213–223.
3. McIntosh SW, de Pontieu B, Carlsson M *et al.* Alfvénic waves with sufficient energy to power the quiet solar corona and fast solar wind. *Nature* 2011; **475**: 477–480.
4. Morton RJ, Sharma R, Tajfirouze E *et al.* Alfvénic waves in the inhomogeneous solar atmosphere. *Reviews of Modern Plasma Physics* 2023; **7**:17.
5. Nakariakov VM, Ofman L, Deluca EE *et al.* TRACE observation of damped coronal loop oscillations: Implications for coronal heating. *Science* 1999; **285**: 862–864.
6. He JS, Tu CY, Marsch E *et al.* Upward propagating high-frequency Alfvén waves as identified from dynamic wave-like spicules observed by SOT on Hinode. *Astronomy and Astrophysics* 2009; **497**: 525–535.
7. Tian H, McIntosh SW, Wang T *et al.* Persistent Doppler Shift Oscillations Observed with Hinode/EIS in the Solar Corona: Spectroscopic Signatures of Alfvénic Waves and Recurring Upflows. *the Astrophysical Journal* 2012; **759**:144.
8. Stangalini M, Consolini G, Berrilli F *et al.* Observational evidence for buffeting-induced kink waves in solar magnetic elements. *Astronomy and Astrophysics* 2014; **569**:A102.
9. Chandrashekhar K, Morton RJ, Banerjee D *et al.* The dynamical behaviour of a jet in an on-disk coronal hole observed with AIA/SDO. *Astronomy and Astrophysics* 2014; **562**:A98.
10. Okamoto TJ, Antolin P, De Pontieu B *et al.* Resonant Absorption of Transverse Oscillations and Associated Heating in a Solar Prominence. I. Observational Aspects. *the Astrophysical Journal* 2015; **809**:71.
11. Gao Y, Tian H, Van Doorsselaere T *et al.* Decayless Oscillations in Solar Coronal Bright Points. *the Astrophysical Journal* 2022; **930**:55.
12. Gao Y, Hou Z, Van Doorsselaere T *et al.* Detection of decayless oscillations in solar transition region loops. *Astronomy and Astrophysics* 2024; **681**:L4.
13. Li D, Hou Z, Bai X *et al.* Simultaneous detection of flare-associated kink oscillations and extreme-ultraviolet waves. *Science in China E: Technological Sciences* 2024; **67**: 1592–1601.

14. Tomczyk S, Card GL, Darnell T *et al.* An Instrument to Measure Coronal Emission Line Polarization. *Solar Physics* 2008; **247**: 411–428.
15. Tomczyk S, McIntosh SW, Keil SL *et al.* Alfvén Waves in the Solar Corona. *Science* 2007; **317**: 1192.
16. Van Doorsselaere T, Nakariakov VM and Verwichte E. Detection of Waves in the Solar Corona: Kink or Alfvén? *the Astrophysical Journal Letters* 2008; **676**: L73.
17. Thurgood JO, Morton RJ and McLaughlin JA. First Direct Measurements of Transverse Waves in Solar Polar Plumes Using SDO/AIA. *the Astrophysical Journal Letters* 2014; **790**:L2.
18. Liu J, McIntosh SW, De Moortel I *et al.* On the Parallel and Perpendicular Propagating Motions Visible inPolar Plumes: An Incubator For (Fast) Solar Wind Acceleration? *the Astrophysical Journal* 2015; **806**:273.
19. Morton RJ, Tomczyk S and Pinto R. Investigating Alfvénic wave propagation in coronal open-field regions. *Nature Communications* 2015; **6**:7813.
20. Weberg MJ, Morton RJ and McLaughlin JA. An Automated Algorithm for Identifying and Tracking Transverse Waves in Solar Images. *the Astrophysical Journal* 2018; **852**:57.
21. Weberg MJ, Morton RJ and McLaughlin JA. Using Transverse Waves to Probe the Plasma Conditions at the Base of the Solar Wind. *the Astrophysical Journal* 2020; **894**:79.
22. Baweja U, Pant V, Krishna Prasad S *et al.* Coexistence of Longitudinal and Transverse Oscillations in Polar Plumes Observed with Solar Orbiter/Extreme Ultraviolet Imager. *the Astrophysical Journal Letters* 2025; **991**:L45.
23. Qi Y, Guo M, Huang Z *et al.* Propagating Kink Waves in Chromospheric Jetlike Structures and Coronal Plumelets. *the Astrophysical Journal* 2026; **1001**:173.
24. Yang Z, Tian H, Tomczyk S *et al.* Mapping the magnetic field in the solar corona through magnetoseismology. *Science in China E: Technological Sciences* 2020; **63**: 2357–2368.
25. Yang Z, Bethge C, Tian H *et al.* Global maps of the magnetic field in the solar corona. *Science* 2020; **369**: 694–697.
26. Yang Z, Tian H, Tomczyk S *et al.* Observing the evolution of the Sun's global coronal magnetic field over 8 months. *Science* 2024; **386**: 76–82.
27. Gao Y, Tian H, Van Doorsselaere T *et al.* Measurements of the Solar Coronal Magnetic Field Based on Coronal Seismology with Propagating Alfvénic Waves: Forward Modeling. *Research in Astronomy and Astrophysics* 2025; **25**:015010.
28. Morton RJ, Weberg MJ and McLaughlin JA. A basal contribution from p-modes to the Alfvénic wave flux in the Sun's corona. *Nature Astronomy* 2019; **3**: 223.
29. Morton RJ, Gao Y, Tajfirouze E *et al.* Evidence for small-scale torsional Alfvén waves in the solar corona. *Nature Astronomy* 2026; **10**: 42–53.
30. Van Doorsselaere T, Srivastava AK, Antolin P *et al.* Coronal Heating by MHD Waves. *Space Science Reviews* 2020; **216**:140.
31. Banerjee D, Krishna Prasad S, Pant V *et al.* Magnetohydrodynamic Waves in Open Coronal Structures. *Space Science Reviews* 2021; **217**:76.
32. Gao Y, Van Doorsselaere T, Tian H *et al.* Propagating kink waves in an open coronal magnetic flux tube with gravitational stratification: Magnetohydrodynamic simulation and forward modelling. *Astronomy and Astrophysics* 2024; **689**:A195.
33. McMurdo M, Ballai I, Verth G *et al.* Driven Phase-mixed Alfvén Waves in a Partially Ionized Solar Plasma. *the Astrophysical Journal* 2025; **988**:50.
34. Tomczyk S and McIntosh SW. Time-Distance Seismology of the Solar Corona with CoMP. *the Astrophysical Journal* 2009; **697**: 1384–1391.
35. Threlfall J, De Moortel I, McIntosh SW *et al.* First comparison of wave observations from CoMP and AIA/SDO. *Astronomy and Astrophysics* 2013; **556**:A124.
36. Withbroe GL and Noyes RW. Mass and energy flow in the solar chromosphere and corona. *Annual Reviews of Astronomy and Astrophysics* 1977; **15**: 363–387.
37. Morton RJ, Weberg MJ, Balodhi N *et al.* Estimating the Poynting Flux of Alfvénic Waves in Polar Coronal Holes across Solar Cycle 24. *the Astrophysical Journal* 2025; **985**:13.
38. De Moortel I and Pascoe DJ. The Effects of Line-of-sight Integration on Multistrand Coronal Loop Oscillations. *the Astrophysical Journal* 2012; **746**:31.
39. Pant V, Magyar N, Van Doorsselaere T *et al.* Investigating "Dark" Energy in the Solar Corona Using Forward Modeling of MHD Waves. *the Astrophysical Journal* 2019; **881**:95.
40. Shi M, Van Doorsselaere T, Antolin P *et al.* Forward Modeling of Simulated Transverse Oscillations in Coronal Loops and the Influence of Background Emission. *the Astrophysical Journal* 2021; **922**:60.
41. Bate W, Jess DB, Nakariakov VM *et al.* High-frequency Waves in Chromospheric Spicules. *the Astrophysical Journal* 2022; **930**:129.
42. Petrova E, Magyar N, Van Doorsselaere T *et al.* High-frequency Decayless Waves with Significant Energy in Solar Orbiter/EUI Observations. *the Astrophysical Journal* 2023; **946**:36.
43. Li D and Long DM. A Statistical Study of Short-period Decayless Oscillations of Coronal Loops in an Active Region. *the Astrophysical Journal* 2023; **944**:8.
44. Lim D, Van Doorsselaere T, Berghmans D *et al.* Characteristics and energy flux distributions of decayless transverse oscillations depending on coronal regions. *Astronomy and Astrophysics* 2024; **689**:A16.
45. Shrivastav AK, Pant V, Berghmans D *et al.* Statistical investigation of decayless oscillations in small-scale coronal loops observed by Solar Orbiter/EUI. *Astronomy and Astrophysics* 2024; **685**:A36.
46. Shrivastav AK, Pant V, Kumar R *et al.* On the Existence of Long-period Decayless Oscillations in Short Active Region Loops. *the Astrophysical Journal* 2025; **979**:6.
47. Chitta LP, van Ballegooijen AA, Rouppe van der Voort L *et al.* Dynamics of the Solar Magnetic Bright Points Derived from Their Horizontal Motions. *the Astrophysical Journal* 2012; **752**:48.
48. Soler R, Terradas J, Oliver R *et al.* Energy Transport and Heating by Torsional Alfvén Waves Propagating from the Photosphere to the Corona in the Quiet Sun. *the Astrophysical Journal* 2019; **871**:3.
49. Khomenko E and Collados M. Heating of the Magnetized Solar Chromosphere by Partial Ionization Effects. *the Astrophysical Journal* 2012; **747**:87.

50. Miriyala H, Morton RJ, Khomenko E *et al.* The Coronal Power Spectrum from MHD Mode Conversion above Sunspots. *the Astrophysical Journal* 2025; **979**:236.
51. Morton RJ, Molnar M, Cranmer SR *et al.* High-frequency Coronal Alfvénic Waves Observed with DKIST/Cryo-NIRSP. *the Astrophysical Journal* 2025; **982**:104.
52. Morton RJ and Soler R. On the Origins of Coronal Alfvénic Waves. *the Astrophysical Journal Letters* 2025; **986**:L6.
53. Lynch BJ, Edmondson JK and Li Y. Interchange Reconnection Alfvén Wave Generation. *Solar Physics* 2014; **289**: 3043–3058.
54. He J, Zhu X, Yang L *et al.* Solar Origin of Compressive Alfvénic Spikes/Kinks as Observed by Parker Solar Probe. *the Astrophysical Journal Letters* 2021; **913**:L14.
55. Wyper PF, DeVore CR, Antiochos SK *et al.* The Imprint of Intermittent Interchange Reconnection on the Solar Wind. *the Astrophysical Journal Letters* 2022; **941**:L29.
56. Yang L, He J, Feng X *et al.* Natural Generation of Alfvén Waves from Three-dimensional Bursty Interchange Magnetic Reconnection in the Solar Corona. *the Astrophysical Journal Letters* 2025; **982**:L25.
57. Chen Y, Peter H, Przybylski D *et al.* Magnetic reconnection sustains the mass budget of the solar wind. *Astronomy and Astrophysics* 2025; **702**:L4.
58. Velli M. On the propagation of ideal, linear Alfven waves in radially stratified stellar atmospheres and winds. *Astronomy and Astrophysics* 1993; **270**: 304–314.
59. Van Doorsselaere T, Li B, Goossens M *et al.* Wave Pressure and Energy Cascade Rate of Kink Waves Computed with Elsässer Variables. *the Astrophysical Journal* 2020; **899**:100.
60. Soler R, Terradas J, Verth G *et al.* Resonantly Damped Propagating Kink Waves in Longitudinally Stratified Solar Waveguides. *the Astrophysical Journal* 2011; **736**:10.
61. Terradas J, Goossens M and Verth G. Selective spatial damping of propagating kink waves due to resonant absorption. *Astronomy and Astrophysics* 2010; **524**:A23.
62. Goossens M, Erdélyi R and Ruderman MS. Resonant MHD Waves in the Solar Atmosphere. *Space Science Reviews* 2011; **158**: 289–338.
63. Tiwari AK, Morton RJ and McLaughlin JA. A Statistical Study of Propagating MHD Kink Waves in the Quiescent Corona. *the Astrophysical Journal* 2021; **919**:74.
64. Morton RJ, Tiwari AK, Van Doorsselaere T *et al.* Weak Damping of Propagating MHD Kink Waves in the Quiescent Corona. *the Astrophysical Journal* 2021; **923**:225.
65. Feigelson ED and Babu GJ. *Modern Statistical Methods for Astronomy* (2012).
